# Vascular endothelial cell injury: causes, molecular mechanisms, and treatments

**DOI:** 10.1002/mco2.70057

**Published:** 2025-01-16

**Authors:** Tian Xia, Jiachi Yu, Meng Du, Ximeng Chen, Chengbin Wang, Ruibing Li

**Affiliations:** ^1^ Department of Laboratory Medicine The First Medical Center of Chinese PLA General Hospital Beijing China; ^2^ Department of Laboratory Medicine Medical School of Chinese PLA Beijing China; ^3^ Department of Clinical Laboratory Huaian Hospital of Huaian City Huaian Jiangsu China

**Keywords:** cardiac I/R injury, diabetic vascular injury, endothelial cell, sepsis

## Abstract

Vascular endothelial cells form a single layer of flat cells that line the inner surface of blood vessels, extending from large vessels to the microvasculature of various organs. These cells are crucial metabolic and endocrine components of the body, playing vital roles in maintaining circulatory stability, regulating vascular tone, and preventing coagulation and thrombosis. Endothelial cell injury is regarded as a pivotal initiating factor in the pathogenesis of various diseases, triggered by multiple factors, including infection, inflammation, and hemodynamic changes, which significantly compromise vascular integrity and function. This review examines the causes, underlying molecular mechanisms, and potential therapeutic approaches for endothelial cell injury, focusing specifically on endothelial damage in cardiac ischemia/reperfusion (I/R) injury, sepsis, and diabetes. It delves into the intricate signaling pathways involved in endothelial cell injury, emphasizing the roles of oxidative stress, mitochondrial dysfunction, inflammatory mediators, and barrier damage. Current treatment strategies—ranging from pharmacological interventions to regenerative approaches and lifestyle modifications—face ongoing challenges and limitations. Overall, this review highlights the importance of understanding endothelial cell injury within the context of various diseases and the necessity for innovative therapeutic methods to improve patient outcomes.

## Graphical abstract

Endothelial cell injury is considered a critical initiating factor in the pathogenesis of various diseases, triggered by multiple factors such as disturbed shear stress and mechanical injury, environmental toxins, metabolic disorders, bacterial/viral infections, and dyslipidemia. These factors significantly compromise vascular integrity and function, lead to mitochondrial dysfunction and oxidative stress, and activate inflammatory responses. This review specifically focuses on endothelial injury in cardiac I/R injury, sepsis, and diabetes. Current treatment strategies include pharmacological interventions, lifestyle modifications, stem cell therapies, and nanoparticle‐based treatments, all of which require further optimization.

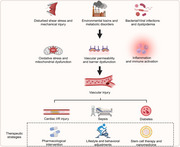

## 1 INTRODUCTION

The arterial wall consists of three distinct layers, arranged in order from the outermost to the innermost: the tunica adventitia, tunica media, and tunica intima. The tunica adventitia is characterized by nerve endings, perivascular adipose tissue, and connective components such as fibroblasts and collagen, all of which contribute markedly to vascular development and remodeling. The tunica media, the intermediate layer, primarily comprises smooth muscle cells (SMCs) that regulate the constriction and dilation of blood vessels. Various mechanical stimuli, including shear stress, pressure, and pharmacological signals, induce the contraction of vascular SMCs by increasing intracellular calcium levels. The innermost layer, the tunica intima, consists of a single layer of endothelial cells (ECs), with an underlying layer of connective tissue. Substances may either pass through the junctions between ECs or be directly absorbed by the cells.^1^ The semi‐permeable endothelial barrier on the inner surfaces of capillaries and venules is critical in regulating the exchange of fluids, electrolytes, and proteins between the blood and surrounding tissues. Maintaining this barrier's integrity is vital for circulatory homeostasis and the proper functioning of various organs.^2^, ^3^, ^4^ Additionally, the endothelium is considered an endocrine organ, participating in numerous paracrine processes by producing and secreting various vasoactive, inflammatory, vasculoprotective, angiogenic, thrombotic, and antithrombotic molecules. Similar to other endocrine tissues, ECs contain receptors that mediate various cellular and hormonal responses, including those related to nitric oxide (NO), protein C, transforming growth factor (TGF), and others.^5^

Endothelial dysfunction (ED) represents a pathological state in which ECs fail to perform their normal physiological roles, thereby impairing their capacity to sustain vascular homeostasis. ED is frequently observed in a range of cardiovascular diseases (CVDs), including hypertension and atherosclerosis, as well as in metabolic disorders such as diabetes and obesity. ECs are pivotal in converting mechanical stimuli, such as shear stress, into intracellular or biochemical signals, which subsequently influence various cellular processes, including proliferation, apoptosis, migration, permeability, remodeling, and gene expression. As a result, ED is often associated with cancer metastasis and inflammatory diseases. The molecular mechanisms underlying ED are multifaceted and are modulated by numerous pathological stimuli, such as low‐density lipoprotein (LDL), reactive oxygen species (ROS), abnormal shear stress, elevated glucose levels, and inflammatory factors. A dysfunctional endothelium exacerbates the production of ROS and enhances vascular inflammation. Inflammatory stimuli activate proinflammatory signaling pathways within ECs, leading to adhesion molecule upregulation. This highlights the interrelationship between OS, inflammation, and ED.^6^, ^7^, ^8^ Additionally, mitochondria serve as the primary sites of ROS generation, and dysfunction of the mitochondrial quality control (MQC) system may function as an upstream regulatory mechanism for ROS production.^9^

This review provides a systematic and comprehensive examination of the causes, molecular mechanisms, clinical relevance, therapeutic interventions, and future directions concerning vascular EC injury. The factors contributing to EC injury are multifaceted, encompassing physical and mechanical forces, chemical and environmental agents, as well as biological and inflammatory mediators. The molecular pathways involved in this injury include OS, mitochondrial dysfunction, endothelial inflammation, immune activation, and disturbances in vascular permeability and barrier integrity. The pivotal role of endothelial injury is emphasized in conditions such as cardiac I/R injury, sepsis, and diabetic vascular complications. In addition, therapeutic strategies are explored, including pharmacological therapies, modifications in lifestyle and behavior, and novel treatments specifically targeting endothelial injury. Last, advancements in developing novel biomarkers and diagnostic tools for assessing EC injury are summarized. This review provides novel insights into vascular injury‐related diseases and proposes innovative approaches for their clinical management.

## 2 THE CAUSES OF EC INJURY

ECs are critical targets in regulating metabolic substances and hemodynamic signals within the microcirculation. In vascular injury, endothelial damage is categorized into external injury and damage induced by circulating substances within the bloodstream. This encompasses physical, mechanical, chemical, and environmental damage, in addition to inflammatory and biological insults. These factors compromise the normal physiological functions of the vascular endothelium. In response to injury, stressed ECs secrete various vasoactive substances, potentially resulting in vasodilation and microcirculatory dysfunction.^10^, ^11^, ^12^ Consequently, this process promotes a shift in EC characteristics from antiadhesive to proadhesive, altering their surface properties from anticoagulant to procoagulant.^13^, ^14^

### 2.1 Physical and mechanical factors

Blood vessels are perpetually subjected to hemodynamic forces, including cyclic stretch and shear stress, due to the pulsatile characteristics of blood flow and pressure. While both ECs and SMCs experience these mechanical forces, shear stress, induced by blood flow, primarily influences the ECs, whereas cyclic stretch, driven by pulsatile pressure, primarily affects the SMCs.^6^ ECs sense variations in wall shear stress and hydrostatic pressure through several surface receptors, including integrins,^15^, ^16^ G‐protein coupled receptors,^17^ membrane lipid rafts,^18^ glycocalyx,^14^ and ion channels.^19^ The activation of these mechanosensors initiates downstream mechanotransduction pathways, which in turn modulate key endothelial functions.^20^ Specifically, changes in shear stress at the cell–cell junctions are recognized by a mechanosensitive complex that includes vascular endothelial cadherin (VE‐cadherin), platelet EC adhesion molecule, and vascular endothelial growth factor receptor 2.^21^

#### 2.1.1 Shear stress and disturbed blood flow

Under physiological conditions, high shear stress is advantageous, as it facilitates adaptive dilation and structural remodeling of the arterial wall via endothelium‐mediated mechanisms. Furthermore, shear stress modulates the growth dynamics of ECs. Evidence suggests that shear stress prevents apoptosis in ECs, a process attenuated by inhibiting NO production. The antiapoptotic effect induced by shear stress is predominantly mediated through the upregulation of endothelial NO synthase (eNOS).^6^ Conversely, in the case of hypertension, prolonged elevation of systemic pressure within the microvasculature accelerates EC aging and turnover. The ability of the endothelium to release endothelium‐derived relaxing factors is compromised, leading to vasoconstriction. Structural alterations in the vascular wall are also induced by hypertension, resulting in modifications within the microcirculatory beds, including remodeling and rarefaction. Of particular significance, remodeling accounts for most of the chronic increase in systemic vascular resistance observed in hypertension. These alterations ultimately result in abnormal shear stress, contributing to EC injury and dysfunction.^22^

#### 2.1.2 Mechanical injury from medical procedures

As open surgical techniques in general surgery, neurosurgery, orthopedics, and advancements in arterial angiography and endovascular procedures have evolved, the incidence of iatrogenic vascular injuries (IVIs) has also increased.^23^ IVIs refer to vascular injuries that occur as a result of surgical procedures, endovascular treatments, radiation therapy, or drug injections. Severe cases of IVIs may even present life‐threatening risks to patients. Initially, endovascular techniques were primarily employed for diagnostic purposes, such as identifying the location of atherosclerotic blockages and monitoring the progression of aneurysms, while surgical interventions remained the main treatment for these conditions. However, with technological progress over the past three decades, many of these lesions can now be treated through minimally invasive endovascular procedures, including balloon angioplasty and stenting. Despite these advancements, balloon dilation and stent placement compromise the integrity of the vascular endothelium, leading to platelet activation, granule release, aggregation, and subsequent thrombosis. Additionally, these procedures can elicit an inflammatory response, promoting the proliferation of vascular SMCs (VSMCs) and increasing the potential for restenosis.^24^ Radiation‐induced vascular injury, first identified over a century ago, continues to present a significant clinical challenge despite notable advancements in radiation oncology. Currently, radiation therapy is used in approximately 50–60% of cancer patients.^25^ The vascular endothelium is particularly vulnerable to ionizing radiation, which can induce ED, characterized by increased permeability, detachment of the basement membrane, and apoptosis.^26^, ^27^

### 2.2 Chemical and environmental factors

In contemporary society, numerous challenges have surfaced as a consequence of rapid economic expansion. Prominent sources of pollution now include the excessive emission of exhaust gases, the pervasive application of nanomaterials, and the production of formaldehyde during home renovations. Furthermore, the prevalence of smoking, secondhand smoke exposure, and unhealthy lifestyle choices has escalated. Metabolic syndrome (MS) has emerged as a critical global public health concern.^28^ Chronic exposure to environmental stressors and poor dietary practices can induce oxidative damage in ECs, which subsequently contributes to the onset of various CV disorders.

#### 2.2.1 Hyperglycemia and metabolic disturbances

MS is increasingly prevalent within the global population. Recent data reveal that MS in China has reached 33.6%.^29^ This rising prevalence has markedly impacted both the health and quality of life of the Chinese population, while also imposing considerable strain on the national economy. MS is characterized by a pathological disruption in the metabolism of proteins, lipids, carbohydrates, and other biochemical substances. It encompasses a complex array of metabolic abnormalities, with central features including obesity, hyperglycemia, dyslipidemia, and hypertension. These factors form the pathological basis for CVDs, diabetes, and certain cancers.^30^, ^31^, ^32^, ^33^, ^34^ Hyperglycemia, a primary pathological hallmark of chronic diabetic complications, directly or indirectly affects EC function. This is mediated through mechanisms such as the activation of the polyol pathway,^35^, ^36^, ^37^, ^38^ the accumulation of advanced glycation end‐products (AGEs),^39^, ^40^, ^41^, ^42^ protein kinase C (PKC) activation,^43^, ^44^, ^45^, ^46^, ^47^ and heightened synthesis of the hexosamine pathway,^48^, ^49^, ^50^, ^51^, ^52^ all of which aggravate vascular complications associated with diabetes. Compared with other traditional risk factors, diabetes has been shown to markedly elevate the risk of CVDs, particularly in conjunction with dyslipidemia. The prevalence of dyslipidemia is markedly higher in diabetic individuals, further exacerbating their susceptibility to CVDs. Diabetes is associated with various complications, including macrovascular disease, particularly diabetes‐induced atherosclerosis, which contributes to cerebrovascular disorders, ischemic heart disease, peripheral arterial disease, and other vascular conditions. These complications are leading causes of mortality among diabetic patients and substantially diminish their quality of life.^53^, ^54^

#### 2.2.2 Environmental toxins

It has been established that particulate matter originating from diesel exhaust and carbon black can enhance the production of ROS, thereby triggering OS responses in ECs. This process involves the activation of cell adhesion molecules, such as intercellular adhesion molecule 1 (ICAM‐1) and vascular cell adhesion molecule 1 (VCAM‐1), which are expressed on the plasma membrane.^55^ Furthermore, silver nanomaterials have been found to elevate ROS production in human umbilical vein ECs (HUVECs), impairing cell proliferation, compromising cell membrane integrity, and inducing significant apoptosis. These nanomaterials also promote the adhesion of a substantial number of monocytes to ECs, potentially increasing the risk of early atherosclerotic development.^56^ Additionally, the expression of fibrinolysis‐related genes and proteins is altered by single‐walled carbon nanotubes, contributing to ED and facilitating thrombogenesis.^57^ Nicotine, the primary toxic component of tobacco, represents another significant factor responsible for endothelial damage. It increases the risk of thrombosis by inducing endothelial injury and dysfunction.^58^ Moreover, the widespread consumption of high‐salt diets has emerged as a potential dietary factor contributing to endothelial injury. High salt intake has been shown to elevate ROS levels in ECs and inhibit NO activity. Studies indicate that salt‐induced OS in cells triggers the reorganization of cytoskeletal proteins in aortic ECs, mediated by the p38‐heat shock protein 27 (HSP27) signaling pathway. This results in a reduced expression of phosphorylated endothelial NOS (P‐eNOS), suppressed NO release, and subsequent EC injury.^59^, ^60^, ^61^, ^62^

### 2.3 Inflammatory and biological factors

Inflammation constitutes the host's defensive immune response to external harmful stimuli. Lipopolysaccharide (LPS), a major component of the bacterial cell wall, is a potent inducer of inflammation and ED in humans.^63^ This response is commonly observed in acute inflammatory conditions, such as septic shock, and in chronic low‐grade inflammatory diseases like rheumatoid arthritis^64^, ^65^ or type 2 diabetes,^66^ where it serves a pivotal function in promoting ED and the progression of atherosclerosis. Furthermore, it has become increasingly evident that, in addition to atherosclerosis, arterial hypertension can also be classified as a low‐grade inflammatory disease.^67^, ^68^ During inflammatory processes, macromolecules and immune cells from the vascular system migrate into the tissue, where they perform their functions, with the vascular endothelium serving as a critical barrier in this process. Numerous factors associated with inflammatory and immune‐mediated vascular diseases have been identified as triggers for EC apoptosis.^69^ Changes in the vascular endothelium are crucial for migrating leukocytes to sites of inflammation. Although these migrated inflammatory cells assist in the clearance of pathogens and foreign particles, they simultaneously contribute to tissue damage.^70^

#### 2.3.1 Infection‐induced inflammatory and immune responses

In bacterial, fungal, or viral infections, both exogenous pathogen‐associated molecular patterns (PAMPs) and endogenous damage‐associated molecular patterns (DAMPs) elicit endothelial activation, disrupting its structural and functional integrity.^71^ Pathogens directly engage ECs via pattern‐recognition receptors (PRRs),^72^, ^73^ leading to the inflammasome assembly. Moreover, pathological stimuli and conditions, including ROS, K^+^ efflux, mechanical stress, and abnormal metabolites, can also trigger PRR activation and inflammasome formation.^74^, ^75^ Additionally, pathogens activate downstream inflammatory signaling pathways, such as the nuclear factor kappa‐B (NF‐κB) and mitogen‐activated protein kinase (MAPK) cascades.^10^, ^11^, ^12^ Activated ECs then initiate a cascade of intracellular signals, which are relayed to the cytoskeleton. These cytoskeletal proteins mediate the transmission of signals through interactions with extracellular adhesion molecules, notably zona occludens 1 (ZO‐1). As a result, these alterations cause dysregulation in adhesion protein functionality, increased centripetal tension within the cells, widening of intercellular gaps, enhanced permeability, and ultimately, the promotion of leukocyte adhesion and the progression of inflammation.^76^, ^77^ ^78^

#### 2.3.2 Inflammation in dyslipidemia and atherosclerosis

Atherosclerosis, a prevalent chronic inflammatory condition affecting the arterial wall, frequently results in disability or even death. In its advanced stages, it is characterized by the formation of lesions and plaque buildup within the arterial intima. These plaques’ subsequent rupture or erosion can provoke thrombotic events, potentially leading to fatal outcomes. The pathogenesis of atherosclerosis is intricate, with its primary components including lipid accumulation in the arterial wall and sustained inflammation.^79^ ROS generated in the body oxidize LDL to form oxidized LDL (oxLDL). oxLDL is internalized by macrophages through scavenger receptors, which leads to the development of lipid‐enriched foam cells, a defining feature of early atherosclerotic lesions. Modified LDL particles, particularly oxLDL, contribute to cellular aging by decreasing NO levels^80^ and can induce OS by activating NF‐κB, thereby fostering ROS accumulation and the increased secretion of the proinflammatory cytokine interleukin‐8 (IL‐8). This process facilitates the adhesion of monocytes to ECs, ultimately resulting in ED. oxLDL is pivotal not only in the initial stages of atherosclerosis but also in the rupture of plaques during later stages.^81^

## 3 MOLECULAR MECHANISMS OF VASCULAR EC INJURY

As has been previously emphasized, endothelial injury is recognized as a hallmark of numerous CVDs. It is regarded as both the initiating factor and a fundamental underlying mechanism in these diseases, serving an indispensable function in their onset and markedly contributing to the progression of organ damage. As a result, considerable efforts have been directed toward identifying the factors and precise mechanisms responsible for endothelial damage, as well as investigating the alterations in active substances—both detrimental and protective—secreted by the injured endothelium, and their involvement in the progression of disease.^82^ Nonetheless, the exact mechanisms responsible for EC injury remain insufficiently understood. During endothelial injury, alterations in vasodilation, irregularities in the production and secretion of active substances, disturbances in energy metabolism, and morphological damage are typically observed. The primary pathophysiological mechanisms implicated include OS and mitochondrial dysfunction, inflammation, and immune activation, along with vascular permeability changes and barrier dysfunction.^83^ Consequently, this section will focus on a comprehensive discussion of the specific molecular mechanisms underlying EC injury.

### 3.1 OS and mitochondrial dysfunction

**FIGURE 1:**
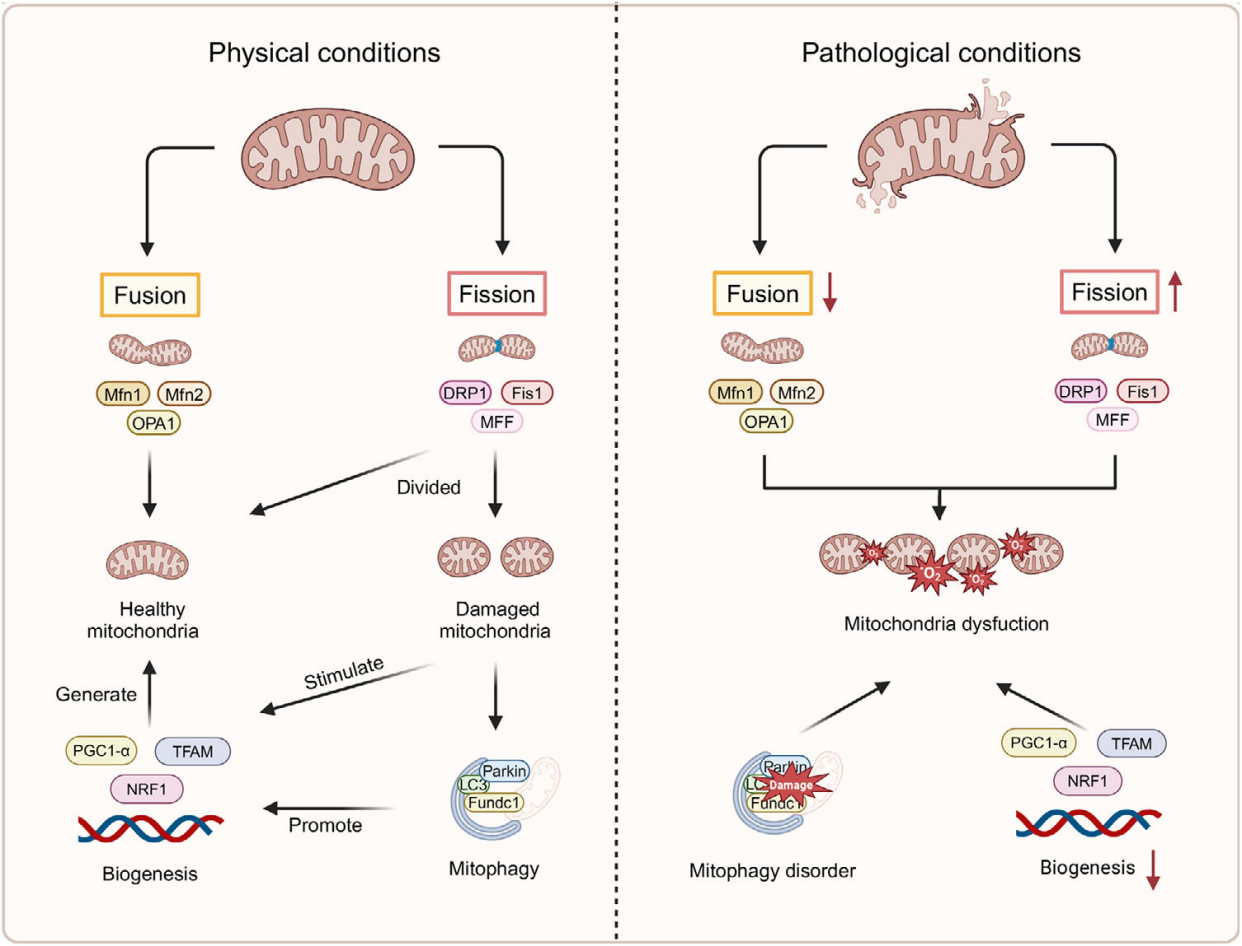
Regulatory mechanisms of MQC. Under normal conditions, dysfunctional mitochondria can fuse with healthy mitochondria or be cleared through mitophagy to restore intracellular balance. And damaged mitochondria are degraded via mitophagy. mitochondrial biogenesis is triggered as a compensatory response to fission and mitophagy. Under pathological conditions, mitochondrial fission increases, fusion decreases, mitophagy becomes dysregulated, and biogenesis is reduced. This leads to a disruption of the MQC system, resulting in mitochondrial dysfunction and excessive production of ROS. Created with www.biorender.com.

Mitochondria, double‐membraned and semi‐autonomous organelles, are found in the majority of eukaryotic cells, occupying approximately one‐third of the total cell volume, and are pivotal in maintaining cellular homeostasis. Their key biological functions include the synthesis of adenosine triphosphate (ATP) via oxidative phosphorylation, as well as the regulation of redox and calcium balance, among other critical roles. In vascular ECs, mitochondria are involved in energy metabolism, ATP production, and regulating diverse cellular signaling pathways and functions.^84^, ^85^, ^86^ Moreover, mitochondria are indispensable for angiogenesis and the secretion of VEGF. Recently, a growing body of research has highlighted the association between mitochondrial dysfunction and endothelial damage, making it a focal point in CVD investigations. MQC encompassing mitochondrial dynamics, mitophagy, and biogenesis, coordinates a hierarchical network of pathways, acting from individual protein molecules to the entire organelle.^87^, ^88^, ^89^ Under normal physiological conditions, the MQC system operates with remarkable precision to eliminate damaged mitochondrial fragments and restore the integrity of the mitochondrial network.^90^, ^91^ However, in pathological states, mitochondrial dysfunction disrupts the balance between mitochondrial fusion and fission, impairs mitophagy, and hinders biogenesis. These dysfunctions result in altered mitochondrial morphology, reduced membrane potential, elevated ROS production, and the initiation of apoptosis. Such alterations are believed to contribute markedly to the pathogenesis of a range of diseases^92^ (Figure 1).

The processes of mitochondrial fission and fusion are fundamental to mitochondrial dynamics, with their balance serving a pivotal function in determining both the morphology and functionality of mitochondria. Mitochondrial fission, essential for removing dysfunctional mitochondria from ECs, is intricately linked to the metabolic demands of the cells.^93^, ^94^ The regulation of mitochondrial fission is predominantly controlled by dynamin‐related protein 1 (DRP1) and its associated receptors, including mitochondrial fission factor, mitochondrial fission protein 1 (FIS1), and mitochondrial dynamics proteins 49/51 kDa (MID49/MID51).^95^ In contrast to mitochondrial fission, mitochondrial fusion involves the integration of various mitochondrial fragments into extended and filamentous structures.^96^, ^97^ Mitochondrial fusion is facilitated by large guanosine triphosphatases (GTPases), such as mitofusin 1 and 2 (MFN1/2) located on the outer mitochondrial membrane, and optic atrophy 1 (OPA1), which mediates the fusion of the inner mitochondrial membrane.^98^ Mitophagy, a selective process of organelle autophagy, serves a critical function in the recycling of misfolded and aggregated proteins, as well as damaged organelles, through lysosomal degradation. This mechanism prevents the accumulation of dysfunctional mitochondria, thereby averting EC injury or death.^99^, ^100^, ^101^, ^102^, ^103^, ^104^, ^105^, ^106^ The mitophagy pathways include PTEN‐induced kinase 1 (PINK1)/parkinson protein 2 (Parkin)‐mediated mitophagy,^107^, ^108^, ^109^, ^110^ as well as receptor‐dependent mitophagy, where key receptors such as FUN14 domain containing 1(FUNDC1), NIP3‐like protein X, and BCL2‐interacting protein 3 play central roles.^111^ Mitochondrial biogenesis, a crucial cellular process, drives the production of new mitochondria, thus enhancing both their quantity and mass to fulfill energy demands.^112^ This process involves the transcription of both mitochondrial and nuclear genomes, the synthesis of lipids and proteins, and assembling these components into a functional respiratory chain.^113^ Central to this process are several key transcription factors, including PPAR‐γ coactivator 1‐α (PGC1‐α), nuclear respiratory factor 1 (NRF1), and mitochondrial transcription factor A (TFAM).^114^

Under pathological conditions, disruptions in MQC are primarily responsible for increased ROS production and ATP depletion, both of which further compromise mitochondrial function and cellular homeostasis. This initiates a detrimental cycle that exacerbates endothelial injury.^115^, ^116^ In particular, the activation of NADPH oxidase (Nox) within ECs leads to the generation of mitochondrial ROS (mtROS), which in turn induces mitochondrial DNA (mtDNA) mutations, causing mitochondrial damage and promoting further mtROS production. This damage accelerates cellular senescence and augments cytoplasmic ROS, resulting in a high‐ROS environment.^117^, ^118^ Excessive ROS production impairs intracellular components, including phospholipids, proteins, and DNA. Oxidation of lipids, proteins, and DNA due to elevated ROS levels can trigger mitochondrial uncoupling and the opening of permeability transition pores, ultimately reducing mitochondrial mass and functionality.^119^, ^120^ NO in ECs can neutralize superoxide and lower ROS levels through the soluble guanylate cyclase–cyclic guanosine monophosphate pathway, promoting vasodilation. However, peroxynitrite, which is produced from the reaction between inducible NOS (iNOS)‐derived NO and superoxide anion, along with uncoupling of eNOS and enzymes such as xanthine oxidase (XO) and lipoxygenase, markedly contribute to ROS generation.^121^, ^122^, ^123^, ^124^, ^125^, ^126^, ^127^, ^128^, ^129^ These processes reduce NO bioavailability and promote vasoconstriction. The downstream effects of ROS in ECs include the activation of NF‐κB, activator protein1 (AP1), hypoxia‐inducible factor (HIF‐1), tumor protein p53 (p53), MAPK, c‐Jun N‐terminal kinase (JNK), and Src kinases,^130^ leading to apoptosis and inflammation.

### 3.2 Endothelial inflammation and immune activation

As previously noted, inflammatory immune responses can activate ECs, leading to cellular damage. Upon infection or tissue injury, ECs are activated, resulting in the upregulation of adhesion molecules and chemokines. These molecules facilitate the recruitment of phagocytes to the site of infection, where ECs contribute to both innate and adaptive immune responses. The resulting increase in vascular permeability promotes the migration of additional immune cells to the affected region. In addition, ECs can function as antigen‐presenting cells by expressing both class I and class II major histocompatibility complex molecules, which allows them to present endothelial‐specific antigens to T cells during inflammation. Toll‐like receptors (TLRs), including TLR2 and TLR4, as well as NOD‐like receptors (NLRs), are also expressed on activated ECs.^131^, ^132^, ^133^ Moreover, the immune system becomes actively engaged, involving dendritic cells, macrophages, neutrophils, T cells, and B cells, which work together to clear pathogens and foreign particles, thereby preserving vascular function.^134^

**FIGURE 2:**
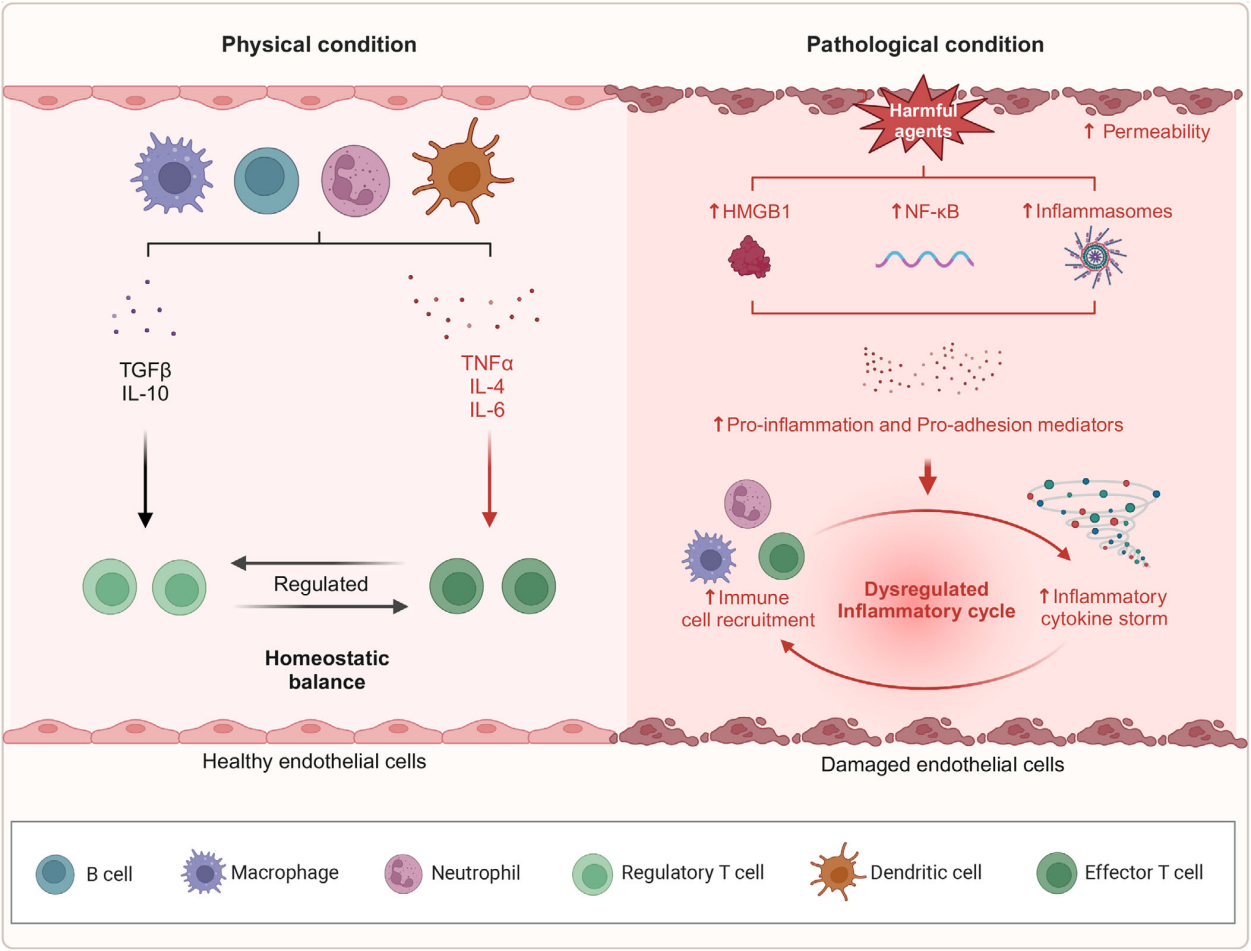
Inflammation. The process of endothelial damage involves an inflammatory response. Under normal conditions, the activation and inhibition of inflammation are balanced, maintaining vascular stability and monitoring pathogen invasion. In pathological states, endothelial cell injury occurs, leading to disruption of the endothelial barrier. Concurrently, factors such as HMGB1, NF‐κB, and inflammasomes are upregulated, promoting the release of inflammatory cytokines and the adhesion of immune cells to eliminate pathogens. However, this heightened inflammatory state exceeds the regulatory capacity of the system, resulting in further damage to endothelial cells. Created with www.biorender.com.

An enhanced understanding of the molecular mechanisms underlying the inflammatory response has emerged, particularly concerning the following functional proteins: (1) *NF‐κB*: This protein serves a pivotal function in endothelial injury‐induced inflammation, serving as a central signaling pathway. Various stimuli activate the NF‐κB signaling cascade, upregulating genes associated with inflammatory cytokines. Recent studies have demonstrated that polychlorinated biphenyls (PCBs) can epigenetically increase the expression of the NF‐κB subunit p65, thereby initiating endothelial inflammatory responses.^135^ (2) *High mobility group protein box 1 (HMGB1)*: HMGB1 is a key inflammatory mediator closely associated with acute coronary syndrome and pulmonary hypertension pathogenesis. Together with heat shock proteins (HSPs) and mtDNA, HMGB1 functions as a DAMP that triggers structural and functional impairments in ECs.^136^ Experimental studies have shown that HMGB1 induces inflammation via the TLR4 and interferon regulatory factor 3 signaling pathways.^137^ (3) *Inflammasomes*: These newly identified multiprotein complexes, with an approximate molecular weight of 100 kDa, are pivotal in the pathogenesis of diseases such as atherosclerosis, cardiac I/R injury, and type 2 diabetes. It is well‐established that the inflammatory cytokine IL‐1β is activated and secreted through inflammasome regulation.^138^ Under normal physiological conditions, immune cells in the vasculature manage inflammation to eliminate pathogens, while anti‐inflammatory cytokines such as IL‐10 and TGFβ^139^, ^140^ help preserve immune homeostasis by inhibiting T cell responses. However, in pathological conditions, harmful agents induce EC injury and increase vascular permeability, which triggers the upregulation of NF‐κB, HMGB1, and inflammasomes. This cascade leads to excessive adhesion of inflammatory cells and the onset of a cytokine storm, thereby disrupting the inflammatory balance (Figure 2).

Recent investigations have elucidated that inflammatory mechanisms are markedly influenced by the regulation of inflammation‐associated microRNAs, including miR‐126, miR‐155, miR‐221/222, miR‐31, miR‐17‐3p, miR‐10a, miR‐663, miR‐125a‐5p, and miR‐125b‐5p. These microRNAs modulate downstream target proteins, such as VCAM‐1, regulator of G‐protein signaling 16, erythroblast transformation‐specific 1, angiotensin II type 1 receptor (AT1R), E‐selectin, ICAM‐1, mitogen‐activated protein kinase kinase kinase 7, and β‐transducin repeat‐containing protein.^141^ In CVDs, EC senescence is frequently observed. During endothelial senescence, a variety of inflammatory mediators, including tumor necrosis factor alpha (TNFα), IL‐1β, IL‐2, IL‐6, IL‐8, C‐C motif chemokine ligand 5 (CCL5), ICAM, and VCAM, are secreted, a process is known as “senescence‐associated inflammation,” which exacerbates EC injury.^83^

### 3.3 Vascular permeability and barrier dysfunction

**FIGURE 3:**
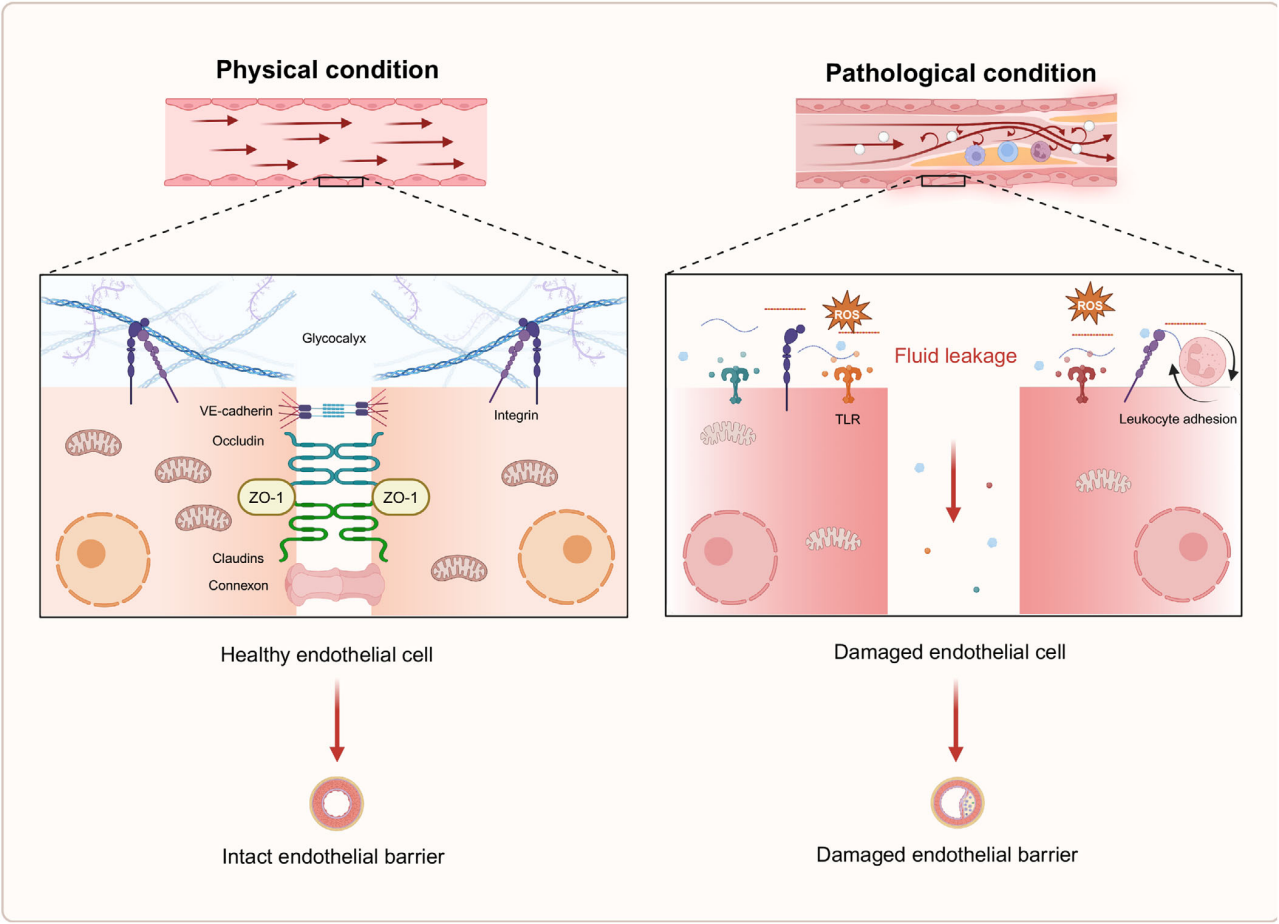
Vascular barrier dysfunction. Vascular permeability is dynamically regulated to maintain tissue homeostasis. The functionality of the vascular endothelial barrier is primarily dependent on the integrity of endothelial cell junctions and the glycocalyx. Barrier damage is typically marked by shedding of glycocalyx components and reconstruction of the surface molecules, which promotes the adhesion of leukocytes. Elevated plasma levels of these components can activate TLR‐4, triggering the NF‐κB pathway and promoting the release of proinflammatory cytokines, further exacerbating endothelial cell injury. Moreover, exposure to harmful factors disrupts intercellular junctions, leading to increased vascular leakage and impaired communication between endothelial cells. Created with www.biorender.com.

Vascular permeability is intricately regulated to preserve tissue homeostasis. In response to the organism's physiological demands, permeability may be modulated by various regulatory mechanisms and stimuli that influence the strength of EC junctions.^142^ The efficacy of the vascular endothelial barrier is predominantly determined by the integrity of EC junctions and the glycocalyx. This glycocalyx, a heparan sulfate (HS)‐enriched layer composed of glycosaminoglycans (GAGs) and proteoglycans, envelops the healthy vascular endothelium. Its highly hydrated and negatively charged structure forms a gel‐like surface, contributing to the barrier's ability to reflect albumin.^143^, ^144^ EC junctions can be classified into three types: adhesive, tight, and gap junctions. Among these, VE‐cadherin is regarded as the principal adhesion protein responsible for maintaining the integrity of the endothelial barrier. VE‐cadherin regulates endothelial cell–cell adhesion and barrier function through four primary mechanisms: phosphorylation of VE‐cadherin and associated proteins, remodeling of VE‐cadherin and actin filaments, internalization of VE‐cadherin, and cleavage of the extracellular domain of VE‐cadherin.^145^, ^146^ Under pathological conditions, damage to the glycocalyx and a reduction in intercellular junctions lead to increased vascular permeability and compromised barrier function (Figure 3).

Glycocalyx damage is typically marked by thinning, degradation, and shedding of its constituent components. Under normal conditions, circulating levels of syndecan‐1 and HS are present in the bloodstream, with their concentrations notably increasing upon glycocalyx injury. Factors such as inflammation, I/R, hypervolemia, and significant cardiac and vascular surgeries have all been implicated in causing glycocalyx disruption.^147^, ^148^ Research indicates that glycocalyx damage can occur as early as 60 min following intestinal ischemia.^149^ During severe hemorrhage, plasma albumin levels drop, often leading to the activation of matrix metalloproteinases (MMP‐9 and MMP‐13). These MMPs cleave the covalent bonds between the extracellular portion of syndecan proteoglycans and GAGs, triggering glycocalyx shedding.^148^ Prolonged ischemia and hypoxia typically result in cellular swelling and necrosis at the ischemic site, which releases xanthine oxidase (XOR) into the circulation. XOR interacts with GAG chains of the glycocalyx, generating substantial amounts of ROS that contribute to glycocalyx degradation. Chappell et al.^150^ demonstrated that, following 20 min of ischemia and subsequent reperfusion in isolated guinea pig hearts, coronary glycocalyx shedding occurred, likely due to OS. Furthermore, glycocalyx damage is an early feature of inflammation. Bacterial LPS and proinflammatory cytokines, such as TNFα, can directly induce glycocalyx shedding. TNFα is known to activate mast cells, promoting their degranulation and the release of various mediators, including cytokines, histamines, proteases, and heparanase, which all contribute to the breakdown of glycocalyx components and the disruption of endothelial integrity.^151^ Elevated plasma levels of glycocalyx components, such as HS, can activate TLR4 on macrophages, triggering the release of additional proinflammatory cytokines, including TNFα and IL‐6, via the NF‐κB signaling pathway, further exacerbating EC injury.

Vascular hyperpermeability is primarily driven by the reorganization of EC junctions, especially tight and adhesive junctions, along with cytoskeletal modifications that generate contractile forces within the cells. These changes lead to cell morphology alterations, contributing to increased permeability.^152^, ^153^, ^154^ Several signaling pathways regulate the mRNA and protein expression of vascular endothelial tight junctions, including PKC, protein kinase A (PKA), protein kinase G (PKG), MAPK, and phosphoinositide 3‐kinase (PI3K)/protein kinase B (Akt). The transcription factors involved in these processes include NF‐κB, snail, kruppel‐like factor 2 (KLF2), and p53.^155^ Under conditions such as inflammation, ischemia, and pharmacological influences, an abnormal distribution of junctional proteins is often observed. One of the most common redistribution mechanisms involves the endocytosis or internalization of junctional proteins from the cell membrane into the cytoplasm.^156^ In brain microvascular ECs, for example, short‐term stimulation with monocyte chemoattractant protein‐1 (MCP‐1) can trigger the endocytosis of claudin‐5 and occludin through a caveolin‐dependent pathway, reducing barrier function. Upon removal of MCP‐1, these proteins are restored to the cell membrane.^157^ Cytokines such as TNFα, IL‐1β, and VEGF can aggravate vascular endothelial leakage by modifying the expression and distribution of tight junction proteins, including claudin‐5, ZO‐1, and occludin.^12^, ^158^ The dissolution of VE‐cadherin and its associated calreticulin complex, as well as stress fiber formation accompanied by increased actin‐myosin contraction, further enhance endothelial permeability.^145^, ^146^ Moreover, ischemic and hypoxic cellular injury alters the expression ratio of junctional proteins, thereby affecting gap junction permeability, endothelial cell‐to‐cell communication, and the overall integrity of the vascular endothelium.

## 4 CLINICAL SIGNIFICANCE OF EC INJURY

EC injury is recognized as a pivotal initiating factor in various CVDs. A comprehensive understanding of its clinical consequences is essential for advancing more effective prevention and therapeutic strategies. Several investigations have employed animal and cellular models to explore the mechanisms underlying EC injury in these conditions. This review examines EC injury in the context of cardiac I/R injury, sepsis, and diabetes.

### 4.1 Cardiac I/R injury

Acute myocardial infarction (AMI) is primarily induced by thrombus formation within the coronary arteries following the rupture of unstable atherosclerotic plaques.^159^ Post‐AMI, thrombolytic therapy, or percutaneous coronary intervention (PCI) remain the most effective methods for minimizing myocardial injury and enhancing clinical outcomes.^160^ However, restoring blood flow to the ischemic myocardium can paradoxically aggravate tissue damage, a phenomenon called I/R injury. The onset of I/R injury markedly reduces the clinical advantages of revascularization, yet its underlying mechanisms and possible therapeutic approaches are still actively under investigation.^161^, ^162^ Cardiac I/R injury is associated with considerable cardiomyocyte death, myocardial stunning, reperfusion arrhythmias, disturbances in coronary microcirculation, and the no‐reflow phenomenon. Studies have indicated that ECs exhibit greater susceptibility to injury during the reperfusion phase than cardiomyocytes.^163^, ^164^ Despite this, current research predominantly investigates cardiomyocyte damage during I/R injury, with a relatively limited focus on EC injury. The ratio of vascular ECs to cardiomyocytes in the heart is approximately 3:1, with the distance between cardiac microvascular ECs and the nearest cardiomyocytes being merely 1–2 µm. Given this structural organization, microvascular ECs not only regulate blood flow in adjacent tissues through the modulation of microvessel diameter but also serve an integral function in cardiac growth, tissue metabolism, contractile function, and rhythm control via paracrine and autocrine signaling mechanisms.^165^

**FIGURE 4:**
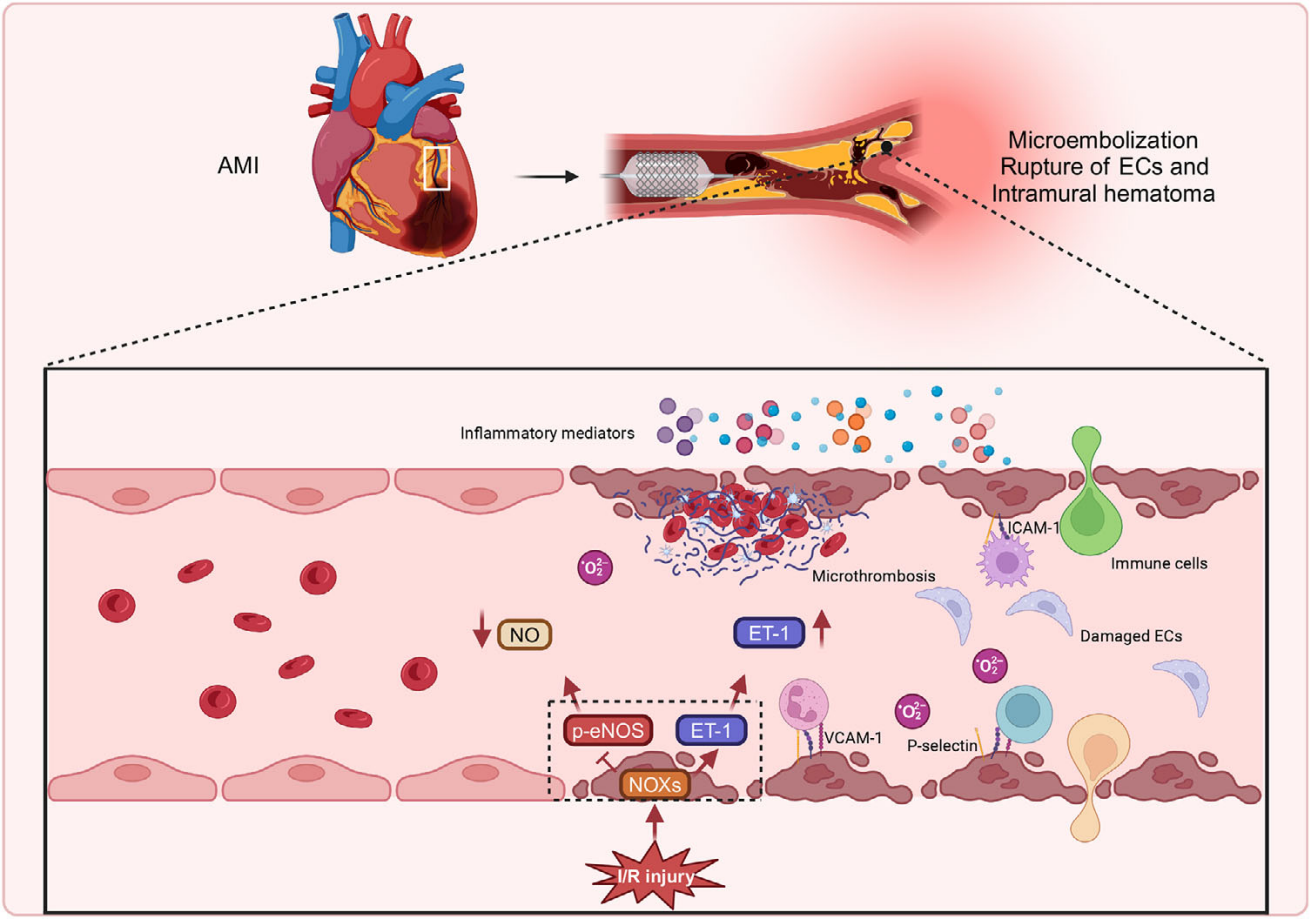
Cardiac I/R injury pathogenesis explicated. Following cardiac I/R, microvascular damage involves complex, multifaceted interactions at the molecular, cellular, and tissue levels. Key features include a reduction in eNOS activity and diminished NO production, along with elevated endothelin‐1 (ET‐1) expression, leading to impaired vasodilation. In parallel, damaged endothelial cells increase the expression of adhesion molecules, chemokines, and cytokines, while the production of superoxide anions rises, promoting leukocyte adhesion and rolling, which intensifies inflammation and thrombosis. These pathological changes further exacerbate cardiac I/R injury and contribute to endothelial cell death. Created with www.biorender.com.

The molecular and cellular mechanisms associated with I/R injury are exceedingly intricate, involving the accumulation of ions, disturbances in energy metabolism, formation of ROS, dysfunction in NO metabolism, immune system activation, autophagy, apoptosis, ED, and damage to the microcirculation.^166^ Among these, ED and microcirculatory damage represent central features and fundamental processes driving I/R injury. Coronary endothelial I/R injury is characterized by: (1) a reduction in eNOS, which correlates with diminished basal NO production; (2) neutrophil adhesion to the coronary endothelium, secondary to the decreased NO levels; (3) enhanced expression of surface‐selective adhesion proteins; and (4) increased production of superoxide anions^167^, ^168^, ^169^ (Figure 4). Electron microscopy conducted during ischemia reveals prominent ultrastructural changes in capillary ECs, suggesting that the morphological alterations of microvascular damage induced by ischemia may serve a direct function in subsequent reperfusion injury.^170^ Moreover, Lu et al.^171^ reported that iodinated n‐butyl fluoropiperidine (F2) mitigates damage to cardiac microvascular ECs during I/R injury through modulation of the ROS/MAPK/early growth response gene 1 pathway.

Conversely, during PCI, balloon dilation or stent deployment may result in thrombi or atherosclerotic material rupture, causing distal microvascular obstruction by particulate matter.^172^ The mechanical injury inflicted upon ECs during the procedure leads to the upregulation of cell adhesion molecules, chemokines, and cytokines, facilitating the recruitment of leukocytes, particularly neutrophils, to the site of injury.^173^ In addition to the obstruction induced by the intervention, reperfusion itself can exacerbate microvascular blockage. Damage to endothelial and myocardial cells during reperfusion further amplifies the inflammatory response, marked by an increase in the expression of L‐selectin ligands in ECs and the release of proinflammatory mediators from myocardial cells, which intensifies the adhesion of neutrophils and monocytes to the endothelium.^174^, ^175^ Simultaneously, ECs activated by immune mediators exhibit heightened coagulation activity, which promotes platelet adhesion, aggregation, and fibrin deposition,^176^ thereby worsening microvascular obstruction. Furthermore, restoring the cardiac microvasculature in patients undergoing PCI largely depends on angiogenesis. In vitro studies have shown that hypoxia/reoxygenation conditions decrease the expression of angiogenesis‐related genes in ECs, impairing their proliferative, migratory, and tube‐forming capabilities.^177^ Minimizing EC injury during I/R injury can foster angiogenesis and improve cardiac function,^178^ suggesting that endothelial injury may impede angiogenesis, potentially contributing to unfavorable outcomes following PCI.

Sustained reperfusion‐induced damage to myocardial tissue and the coronary vasculature represents a multifactorial and intricate process.^159^ In comparison with myocardial injury, the disturbances in coronary circulation induced by I/R have historically been underexplored.^118^, ^179^, ^180^ Nevertheless, as awareness of this issue increases, a more profound understanding of the mechanisms driving I/R‐induced EC injury is expected to lead to novel therapeutic approaches for patients affected by I/R injury. A summary of the most recent animal and cell‐based studies concerning cardiac I/R‐induced EC injury is depicted in Table 1.

**TABLE 1:** The latest animal and cell‐based studies related to cardiac I/R‐induced endothelial cell damage.

Species/cell type	Model	Target point	Mechanism	References
Rat and H9C2	Rat: I/R injury (45 min/1 h) H9C2: H2O2	Inflammation	TUG1 silencing attenuates cardiac vascular I/R injury by reducing NF‐κB/p65 expression.	^181^
Mice and HUVECs	Mice: I/R injury (45 min/1h) HUVECs: cultured under hypoxia with the Esumi ischemic buffer	Angiogenesis	FNDC4 alleviates cardiac I/R injury through increasing the expression and secretion of FGF1 from cardiomyocytes to enhance angiogenesis in a paracrine manner.	^182^
Mice and PHECs	Mice: I/R injury (45 min/6h) PHECs: H/R injury	MQC	EC‐S1PR2 initiates excessive mitochondrial fission and elevates ROS production via RHO/ROCK1/DRP1 pathway, exacerbating inflammation and I/R injury.	^183^
Rat CMECs and H9C2	Rat CMECs: hypoxia culture H9C2: H/R	Cell interaction	The miR‐210‐3p delivered by H‐exo inhibits TFR expression by directly interacting with TFR mRNA, resulting in the promotion of cell proliferation and the attenuation of cell ferroptosis in H/R‐treated H9C2 cells.	^184^
Rats and primary CMECs	Rats: I/R injury(30 min/24 h) Primary CMECs: H/R with ischemic buffer	Pyroptosis	KDM3Aactivates the PI3K/Akt signaling pathway and ameliorated I/R‐mediated CMEC pyroptosis, thereby alleviates CMEC I/R injury.	^185^

Abbreviations: H9C2, rat cardiomyocyte cell line; TUG1, taurine‐upregulated gene 1; FNDC4, fibronectin type III domain containing 4; FGF1, fibroblast growth factor 1; PHECs, primary heart endothelial cells; H/R, hypoxia/reoxygenation; S1PR2, sphingosine‐1‐phosphate receptor 2; RHO:Rho family GTPase; ROCK1, Rho‐associated coiled‐coil containing protein kinase 1; H‐exo, hypoxia‐conditioned CMECs‐derived Exo; KDM3A, Lysine‐specific demethylase 3A; CMECs, cardiac microvascular endothelial cell.

### 4.2 Sepsis

**FIGURE 5:**
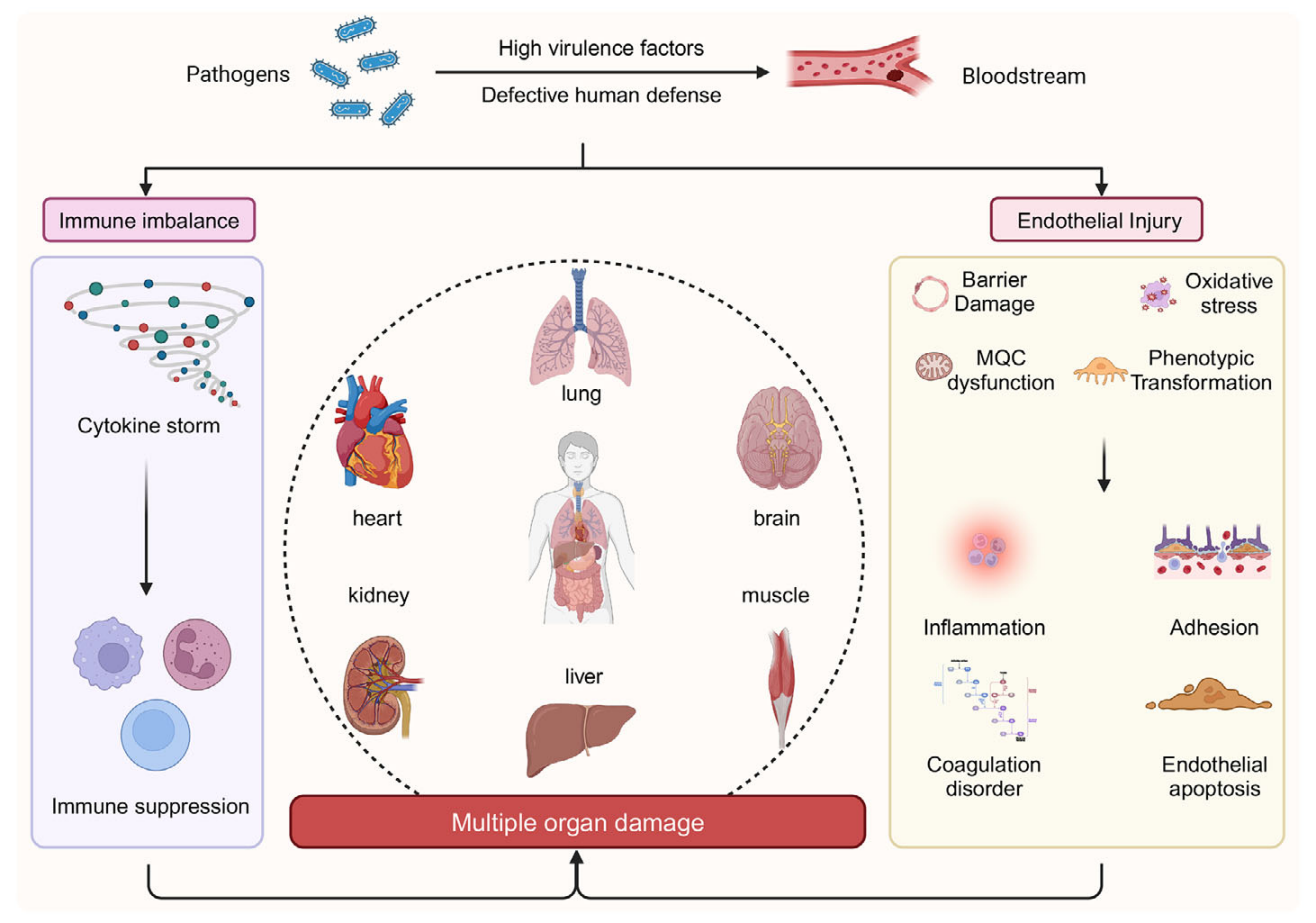
Sepsis pathogenesis explicated. In sepsis, pathogen invasion triggers an excessive production of inflammatory mediators, leading to a cytokine storm, with survivors often entering a paradoxical state of immune suppression. Throughout this process, endothelial cells endure significant damage, including barrier disruption, oxidative stress, mitochondrial dysfunction, and phenotypic changes. These injuries drive coagulopathy, uncontrolled inflammation, and increased vascular permeability. The breakdown of endothelial function typically signals the onset of multiple organ dysfunction syndrome (MODS). Created with www.biorender.com.

Sepsis is defined by life‐threatening organ dysfunction resulting from a dysregulated host response to infection, which is characterized by excessive inflammation alongside immune suppression.^186^ Recent findings have demonstrated that ED is integral to both the onset and progression of sepsis, serving as a significant risk factor for organ dysfunction in septic patients.^187^ It has been observed that systemic endothelial activation is notably more pronounced in septic individuals than in nonseptic counterparts, as indicated by plasma biomarkers.^188^, ^189^ During sepsis, the overproduction of endotoxins and inflammatory cytokines directly compromises EC integrity. Furthermore, inflammatory mediators promote a substantial influx of extracellular Ca^2+^ through pathways such as the LPS/TLR4 signaling cascade^190^ and the G protein signaling pathway.^191^ Endothelial injury, on the one hand, activates inflammatory and coagulation pathways, leading to widespread microthrombus formation and subsequent organ damage. On the other hand, elevated endothelial permeability induces capillary leakage, worsening tissue and organ hypoperfusion and ultimately resulting in multiple organ dysfunction.^192^ Consequently, EC injury and microcirculatory disturbances are pivotal in the progression of multiple organ dysfunction, and enhancing endothelial function is of paramount clinical significance in mitigating sepsis‐related mortality (Figure 5).

Clinical, animal, and cellular investigations have demonstrated that sepsis can lead to the shedding of the endothelial glycocalyx, particularly in smaller vessels. This process is primarily triggered by inflammatory mediators such as TNFα, which activate heparanase and MMP‐9, facilitating the cleavage of extracellular portions of HS proteoglycans and glycoproteins.^14^, ^193^, ^194^, ^195^, ^196^, ^197^, ^198^, ^199^, ^200^ Disruption of the glycocalyx layer impairs barrier function, losing critical signaling molecules and enzymes, diminishing its protective role, and exposing receptors responsible for leukocyte adhesion.^193^ Following acute injury, the restoration of the glycocalyx layer requires approximately 5–7 days, with complete recovery of hydrodynamic activity taking a longer period.^201^ This delayed recovery may partially account for the persistent microcirculatory blood flow impairment observed in septic shock patients, even after resuscitation and optimization of microcirculatory hemodynamics.^202^, ^203^ Concurrently, the upregulation of adhesion molecules on the endothelial surface promotes the recruitment of leukocytes,^204^, ^205^ particularly within the venous segments of the microcirculatory network. Elevated levels of shed adhesion molecules in plasma, such as E‐selectin, VCAM‐1, and ICAM‐1, in sepsis patients are indicative of the severity of organ failure, as supported by in vitro cellular models.^206^, ^207^ Both experimental and clinical studies suggest that modulating the expression of adhesion molecules may offer a potential therapeutic approach for sepsis.^208^ However, complete inhibition may have detrimental effects, as leukocyte infiltration is crucial for microbial clearance and prevention of pathogen spread.^209^

During the inflammatory response, neutrophils, once activated, release ROS and RNS. Simultaneously, the body releases a significant quantity of inflammatory mediators and cytokines, such as IL‐1, interferon, and TNFα. These cytokines either directly or through signal transduction pathways, modulate the phosphorylation state of occludins, claudins, and other cytoskeletal proteins, thus contributing to the reprogramming of ECs toward a secretory phenotype.^76^, ^77^ The phenotypic shift in ECs exacerbates leukocyte adhesion and the secretion of additional inflammatory mediators, ultimately leading to an uncontrolled cytokine storm.^13^ Furthermore, elevated levels of inflammatory cytokines and ROS heighten endothelial damage, inducing apoptosis by activating the B cell lymphoma 2 (BCL2) family or binding to death receptors, which initiates the apoptotic cascade.^210^ Additionally, disruption of the MQC system further promotes ROS production. In sepsis, EC mitochondrial fission is activated, with LPS exposure leading to a concurrent increase in DRP1 and FIS1 expression, resulting in the formation of fragmented mitochondria with a punctate distribution.^211^, ^212^, ^213^ LPS‐induced upregulation of mitophagy in ECs is indicated by a reduction in mitochondrial mass and an increase in PINK1 and Parkin^214^ expression. LPS also impacts mitochondrial biogenesis through 5' AMP‐activated protein kinase (AMPK) signaling.^215^ These changes collectively lead to mitochondrial morphology and dysfunction alterations, thereby increasing the OS burden.

The pathogenesis of sepsis is a highly intricate process, with both clinical and conventional laboratory tests exhibiting notable limitations. Dysfunction of ECs represents a pivotal mechanism in the progression of sepsis. Targeting ECs with therapeutic interventions may offer substantial therapeutic potential. A summary of the most recent animal and cell‐based studies concerning sepsis‐induced EC injury is depicted in Table 2.

**TABLE 2:** The latest animal and cell‐based studies related to sepsis‐induced endothelial cell injury.

Species/cell type	Model	Target point	Mechanism	References
Mice and CMECs	Mice: LPS abdominal injection CMECs: LPS treatment	Inflammation	BMP10 knockdown blocks SIMI progression by inhibiting the HIF‐1α pathway.	^216^
Primary human ECs	LPS treatment	Oxidative stress	ADGRL2 preserves eNOS activity by shifting its binding from caveolin‐1 to heat shock protein 90 and enhances antioxidative responses by increasing NRF2 activity.	^217^
Mice and PAECs	Mice: CLP PAECs: LPS treatment	MQC	SIRT3 deficiency facilitates mtROS production and cytosolic release of mtDNA by impaired Parkin‐dependent mitophagy, promoting to lung ECs pyroptosis through the NLRP3 inflammasome activation.	^218^
Mice and HMEC‐1	Mice: CLP HMEC‐1: LPS treatment	Pyroptosis	Apelin is confirmed to alleviate siALI partially by modulating SIRT1/NLRP3 pathway to inhibit EC pyroptosis.	^219^
Mice and PVECs	Mice: CLP PVECs: LPS treatment	PANoptosis	Lactic acidemia promotes macrophage‐derived eCIRP release, which, in turn, mediates ZBP1‐dependent PVEC PANoptosis in siALI.	^220^

Abbreviations: SIMI, sepsis‐induced myocardial injury; ADGRL2, adhesion G protein‐coupled receptor L2; PAECs, primary pulmonary endothelial cells; CLP, cecal ligation puncture; SIRT3, sirtuin 3; HMEC‐1, human microvascular endothelial cell line 1; siALI, sepsis‐induced ALI; PVECs, pulmonary vascular endothelial cells; eCIRP, extracellular cold‐inducible RNA‐binding protein; ZBP1, Z‐DNA binding protein 1.

### 4.3 Diabetic vascular injury

Diabetes can cause damage to various organs, resulting in a significant number of deaths annually due to both acute and chronic complications associated with the disease. The vascular complications related to diabetes are categorized into macrovascular and microvascular types. Macrovascular complications involve atherosclerotic lesions predominantly affecting large and medium‐sized arteries, such as the coronary arteries and are associated with high morbidity and mortality rates. Atherosclerosis commonly develops at sites of hemodynamic disturbances, characterized by macrophage foam cell formation, EC lesions, and SMC damage.^221^, ^222^, ^223^ On the other hand, diabetes‐related microvascular complications primarily target arterioles and venules, resulting in microcirculatory dysfunction and thickening of the microvascular basement membrane.^224^ Microvessels, primarily composed of ECs, are the first and most vulnerable to damage under hyperglycemic conditions, as they are in direct contact with blood. These microvascular complications include diabetic cardiomyopathy, nephropathy, retinopathy, and other associated conditions, which markedly diminish patients’ quality of life and shorten their lifespan. Early and aggressive interventions aimed at addressing metabolic abnormalities have the potential to improve prognosis.^225^, ^226^

**FIGURE 6:**
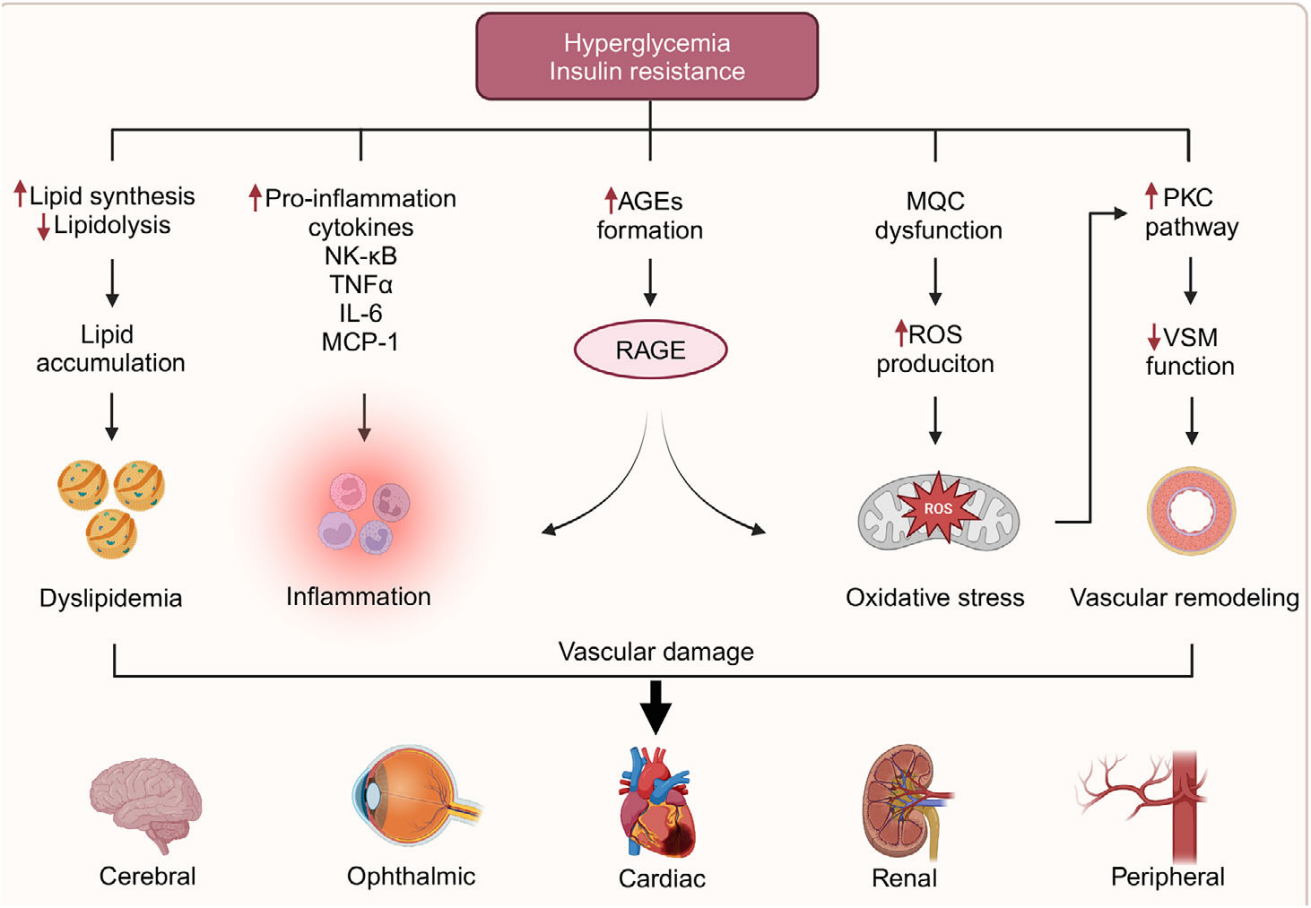
Diabetic vascular injury explicated. Diabetic vascular injury involves the cardiac, cerebral, renal, ophthalmic and peripheral systems. The mechanisms are complex, hyperglycemia and insulin resistance contribute to mitochondrial dysfunction, oxidative stress, and disruptions in metabolic pathways, including the formation of AGEs and activation of the PKC pathway. These interconnected processes work synergistically, ultimately leading to vascular remodeling and functional impairment. Created with www.biorender.com.

The mechanisms through which diabetes induces vascular endothelial damage have attracted considerable attention, particularly concerning disruptions in endothelial metabolism, glycotoxicity, lipotoxicity, and mitochondrial dysfunction, all contributing to the generation of OS and inflammation. In patients with diabetes, microvascular functional changes typically precede significant macrovascular damage.^227^ Research has shown that as glucose levels rise, ECs gradually lose their original morphology, accompanied by a decrease in proliferative capacity.^228^ Insulin resistance, along with diminished insulin secretion, promotes lipid synthesis in hepatocytes and lipolysis in adipocytes, resulting in elevated concentrations of circulating fatty acids and triglycerides.^229^, ^230^ The primary metabolites of diabetic glycotoxins are AGEs, which bind to their cellular receptors (RAGEs), stimulating the production of ROS, NF‐κB, and proinflammatory cytokines such as TNFα, IL‐6, and MCP‐1. This cascade triggers the intracellular generation of excessive ROS, leading to a cycle of OS and inflammation.^231^, ^232^ PKC, an effector in the G protein‐coupled receptor system, is pivotal in maintaining VSMC tone. PKC activation, induced by an excess of ROS and AGEs, results in impaired VSMC function (Figure 6) Hyperglycemia also activates several biochemical pathways, including JNK/stress‐activated protein kinase, p38 mitogen‐activated protein kinase, and the hexosamine pathway. The activation of these pathways contributes to cellular injury, ultimately exacerbating diabetic complications.^233^

Recent studies have notably established that hyperglycemia induces endothelial mitochondriopathy.^234^, ^235^ Mitochondria, impaired by dysfunctional mitophagy or abnormal biogenesis, exhibit a marked susceptibility to excessive ROS production, even without hyperglycemia. Clinical investigations involving diabetic patients undergoing coronary artery bypass grafting have demonstrated mitochondrial morphological abnormalities, such as vacuoles and swollen mitochondria, in poorly controlled individuals (HbA1c > 7.5%), whereas no such abnormalities were observed in well‐controlled patients (HbA1c = 4.4–6.2%) or nondiabetic individuals.^236^ In a similar vein, diabetic patients exhibited increased mitochondrial fragmentation, reduced mitochondrial networking, and heightened expressions of FIS1 and DRP1 in venous ECs.^235^ Additionally, the levels of Ser600‐phosphorylated DRP1 were found to be elevated in endothelial progenitor cells (EPCs) from diabetic patients compared with nondiabetic controls.^237^ In both cellular and animal models, hyperglycemia has been shown to activate DRP1 expression through the JNK‐related signaling pathway, thereby contributing to mitochondrial fission and subsequent EC injury.^238^ In high‐glucose‐treated HUVECs, significant downregulation of PINK1, Parkin, LC3II, Beclin‐1, and autophagy‐related gene 5 was observed, while p62 levels were notably upregulated.^239^ Furthermore, another investigation revealed that in rats with poor glycemic control, there was a significant reduction in the mtDNA copy number, the transcription of biogenesis‐related genes such as PGC1‐α, NRF1, and TFAM, as well as the levels of TFAM bound to mitochondria in the retina, when compared with rats with well‐controlled glycemia.^240^

Coronary vascular ED serves a dual function in diabetes, serving both as a contributor and as a consequence of the disease. It can exacerbate diabetes‐related microvascular and macrovascular complications by fostering vasoconstrictive, proinflammatory, and prothrombotic responses. However, clinically effective treatments for diabetic vascular disease remain absent. The most recent findings from animal and cell‐based studies on diabetic EC injury are summarized in Table 3.

**TABLE 3:** The latest animal and cell experiments related to diabetic endothelial cell damage.

Species/cell type	Model	Target point	Mechanism	References
Mice and HGECs	Mice: intraperitoneally injected with low‐dose streptozotocin HGECs: C‐6 treatment	Inflammation	KLF2 overexpression effectively induces downstream Nos3 expression and suppresses NF‐kB activation in glomerular endothelial cells.	^241^
Rats and HREC	Rat: high fat and sugar diets for 3 weeks and streptozotocin injection HREC: streptozotocin treatment	Inflammation	Increased IS significantly contributes to retinal microvascular damage in DR by inducing the expression of COX‐2 and the production of PGE2.	^242^
HUVECs	Cultured under high glucose conditions	Oxidative stress	Overexpression of ZIPK combines with STAT5A silencing attenuates glucose‐induced alterations in p53 and NOS2 expression, thereby preventing cell damage.	^243^
Rats and primary RVECs	Rats: intraperitoneally injected with streptozotocin RVECs: cultured under high glucose conditions	Methylation	YTHDC1‐mediated m6A methylation regulates diabetes‐induced RVECs dysfunction, YTHDC1‐CDK6 signaling axis could be therapeutically targeted for treating DR.	^244^
Mice and HUVECs	Mice: HFD and intraperitoneally injected with 20 % glucose solution HUVECs: treated with PA‐HG	Pyroptosis	PA‐HG induces the lower expression of SIRT2, leading to the deacetylation of NLRP3 and finally triggering pyroptosis in HUVECs, this can be mitigated by 1,8‐cineole.	^245^

Abbreviations: HGECs, human glomerular endothelial cells; C‐6, novel KLF2 activator compound 6; Nos3, nitric oxide synthase 3; HREC, human retinal endothelial cells; IS, indoxyl sulfate; DR, diabetic retinopathy; COX‐2, cyclooxygenase‐2; PGE2, prostaglandin E2; ZIPK, zipper‐interacting protein kinase; STAT5A, signal transducer and activator of transcription 5A; RVECs, retinal vascular endothelial cells; YTHDC1, YTH domain containing 1; CDK6, cyclin‐dependent kinase 6; HFD, high‐fat and high‐glucose diet; PA‐HG, palmitic acid‐high glucose.

## 5 THERAPEUTIC STRATEGIES FOR VASCULAR EC INJURY

With advancing research, an increasing recognition of the pivotal role of vascular endothelial injury in the pathogenesis of numerous diseases has emerged. Approaches aimed at targeting vascular ECs to enhance endothelial barrier function present promising therapeutic strategies for conditions such as I/R injury, sepsis, atherosclerosis, and diabetes. Contemporary treatment strategies predominantly encompass pharmacological interventions, lifestyle modifications, and novel emerging therapies, all contributing to fresh perspectives in managing these diseases.

### 5.1 Pharmacological intervention

#### 5.1.1 Antioxidants

Given the profound influence of OS on the onset and progression of CVDs, recent investigations have increasingly centered on identifying effective treatment strategies leveraging the antioxidant properties of various compounds.^246^ However, attempts to directly target OS and lipoprotein oxidation have often yielded unsatisfactory outcomes. Despite using a range of antioxidants, clinical trials have failed to demonstrate significant therapeutic benefits. Vitamins C, D, and E, along with beta‐carotene, have shown no efficacy in reducing adverse events in well‐controlled and sufficiently powered studies.^247^, ^248^, ^249^ Consequently, the research focus has shifted from systemic antioxidants to more precise therapies that specifically target localized OS at the site of EC injury. Another key avenue of exploration is identifying natural compounds suitable for long‐term use with minimal adverse effects. Antioxidant precursors, such as N‐acetylcysteine and polyphenolic compounds, have shown potential as therapeutic agents. Moreover, food products and dietary supplements containing these substances—such as black and green tea, red wine, and extra virgin olive oil—are also under investigation.^250^ The antiatherogenic properties of N‐acetylcysteine, polyphenols, flavonoid conjugates, and isoquercetin warrant further research. Notwithstanding, promising findings have been reported for herbal preparations rich in isoflavonoids.^251^

#### 5.1.2 Anti‐inflammatory agents

Inflammation has been identified as an independent risk factor in the progression of CVDs.^252^ Established medications, such as angiotensin‐converting enzyme (ACE) inhibitors, angiotensin II type‐1 (AT‐1) receptor antagonists, statins, and various other CV drugs, exhibit pleiotropic anti‐inflammatory and antioxidant effects, which contribute to the improvement of endothelial function.^253^, ^254^, ^255^, ^256^ Several clinical trials are currently investigating whether anti‐inflammatory treatments can enhance CV outcomes, such as methotrexate therapy (in the phase of the thrombosis, inflammation, and vascular risk trial and CV inflammation reduction trial), which has transformed the practice of rheumatology,^257^, ^258^ and blockade of the cytokine IL‐1β with canakinumab for the management of CV disease (in the phase of canakinumab anti‐inflammatory thrombosis outcomes study).^259^ Moreover, a novel anti‐inflammatory approach could involve the restoration of a disrupted glycocalyx, which serves a pivotal function in increasing susceptibility to atherosclerosis triggers and leukocyte/platelet adhesion.^260^, ^261^, ^262^ Colchicine, another agent frequently utilized for treating inflammatory conditions, has also demonstrated potential in combating CV inflammation. It has become a standard treatment for pericarditis, offering an alternative to glucocorticoid therapy, which was previously hindered by the corticosteroid rebound phenomenon.^263^

#### 5.1.3 Agents targeting endothelial function

An alternative approach to improving endothelial function involves the restoration of cellular redox equilibrium and the elevation of NO levels via the exogenous supplementation of NO donors, such as organic nitrates. Clinically, organic nitrates are utilized to alleviate coronary artery constriction commonly associated with atherosclerosis and ED.^264^ Research has demonstrated that animals treated with isosorbide or pentaerythrityl tetranitrate experienced a reduction in the progression or, in some instances, even regression of atherosclerotic lesions.^265^, ^266^ However, in recent years, the hypothesis that organic nitrates can restore vascular homeostasis has been refuted. Chronic administration of conventional nitrates appears to have a neutral impact or, in certain cases, even exacerbates OS rather than mitigating it.^267^, ^268^, ^269^ Currently, the exogenous administration of L‐arginine has been suggested as a method to recouple eNOS activity and enhance NO bioavailability. By increasing cellular L‐arginine concentrations, this strategy promotes the competition between L‐arginine and asymmetric dimethylarginine (ADMA) for the eNOS active site, thereby attenuating the inhibitory effects of ADMA on NO synthesis.^270^ Furthermore, it is paramount to implement early interventions to prevent or minimize the damage and shedding of the endothelial glycocalyx upon exposure to injurious factors. For instance, sulodexide, an extract derived from porcine intestinal mucosa, possesses anti‐inflammatory properties. Following oral administration, sulodexide is degraded in the gastrointestinal tract into N‐acetyl‐glucosamine moieties, which serve as precursors for synthesizing GAG chains, thereby preserving the endothelial glycocalyx.^271^, ^272^ Additionally, Jacob et al.^273^ observed that serum albumin can reduce I/R injury‐induced damage and shedding of the glycocalyx, alleviate interstitial edema and leukocyte adhesion in the coronary arteries, and partially restore the glycocalyx's barrier function.

### 5.2 Lifestyle and behavioral adjustments

#### 5.2.1 Diet and exercise in preventing ED

Numerous studies have indicated that lifestyle modifications, including physical activity and dietary management, can markedly enhance endothelial function. Exercise, particularly aerobic types, has been shown to improve vascular endothelial function under both pathological and nonpathological conditions, thereby serving as a preventive and therapeutic measure for CVDs. As such, exercise is regarded as an effective nonpharmacological intervention for reducing the prevalence of CVDs. One of the most compelling findings supporting exercise‐induced improvement in vascular function is the enhancement of endothelium‐dependent vasodilation. This is attributed to exercise‐induced alterations in blood flow, which elevate shear stress—a mechanical stimulus that activates potassium channels, facilitating calcium influx into ECs, thereby activating eNOS and increasing NO production.^274^ Furthermore, exercise promotes the proliferation, migration, and differentiation of EPCs into mature ECs, thus facilitating angiogenesis.^275^ Dietary control represents another crucial and effective strategy for improving CV function. Research has demonstrated that high‐fat diets lead to ED, whereas low‐calorie diets not only promote weight loss but also reduce inflammatory factor secretion and increase flow‐mediated dilation (FMD) in the vascular endothelium.^276^, ^277^ However, the long‐term sustainability of dietary control alone is challenging, particularly in obese individuals, for whom comprehensive lifestyle modifications are considered the most effective and enduring approach.^278^

#### 5.2.2 Smoking cessation and pollution reduction strategies

Smoking, widely acknowledged as a risk factor for CVDs, is strongly linked to both the incidence and mortality of CV conditions and exerts a detrimental effect on long‐term prognosis.^279^ It has been established that both short‐term and long‐term cessation of smoking lowers the risk of developing CVDs.^280^ A longitudinal investigation evaluating the effects of smoking and smoking cessation on inflammatory markers associated with CVDs revealed that white blood cell counts were markedly reduced in smokers 1 year following cessation compared with when they were actively smoking.^281^ Smoking cessation has also been shown to exert beneficial effects on endothelial function. A prospective clinical trial conducted internationally involving 1504 current smokers, of whom 36.2% had quit smoking after 1 year, found that although FMD markedly increased after 1 year of cessation (with a decrease in FMD indicative of ED), no notable change in FMD was observed among those who continued smoking. After adjusting for relevant CV risk factors, the improvement in FMD among those who had quit smoking was deemed significant, indirectly suggesting that smoking cessation can enhance CV endothelial function and reduce the incidence of CVDs.^282^ Furthermore, exposure to air pollution also contributes to an increased risk of CVDs,^282^ underscoring the necessity for occupational and environmental health authorities to implement policies to reduce air pollution to mitigate the incidence of CV conditions.

### 5.3 Novel therapeutic approaches

#### 5.3.1 Gene therapy targeting ECs

Gene therapy entails the transfer of therapeutic target genes into relevant organs or tissues, aiming to treat diseases by repairing or replacing genes, thereby facilitating their appropriate expression in somatic cells.^283^ NOS predominantly exists in three isoforms: neuronal (nNOS), iNOS, and eNOS. Yatera et al.^284^ demonstrated that mice with single, double, or complete knockout of all three NOS genes developed severe lipid metabolic disorders, atherosclerosis, and sudden cardiac death after being fed a high‐fat Western diet, highlighting the pivotal role of the endothelial NOS system in maintaining lipid homeostasis. As such, NOS represents a promising target for gene therapy. Concurrently, emerging evidence indicates that epigenetic pathways, regulated by redox processes, markedly contribute to CVDs by directly modulating endothelial function.^285^, ^286^ For example, targeting Nox4 with miR‐146a has reduced ROS levels, potentially mitigating ED.^287^ In contrast, miR‐200 has been found to elevate OS and promote EC senescence.^288^ Furthermore, endothelial function can be enhanced by using DNA (cytosine‐5)‐methyltransferase 1 (DNMT) inhibitors. These inhibitors reverse the silencing of the atheroprotective transcription factor KLF4 in human aortic ECs and adult swine aortas, which is induced by disturbed blood flow. DNMT inhibitors also help restore KLF2 expression in HUVECs, a gene typically repressed in dysfunctional endothelium due to LDL‐mediated DNA methylation.^289^

#### 5.3.2 Regenerative medicine and stem cell therapy

Tissue regeneration mediated by somatic stem/progenitor cells has been recognized as a crucial system for maintaining or restoring the function of various adult organs. Based on their regenerative potential, these stem/progenitor cells are regarded as central to therapeutic strategies for organ repair. Research has demonstrated that cell therapy using culture‐expanded EPCs can promote neovascularization in ischemic tissues, even without angiogenic growth factors. This “supply‐side” approach to therapeutic neovascularization, in which the therapeutic agent is the cellular substrate (EPCs/ECs) rather than the growth factor ligand, was first established by intravenously transplanting human EPCs into immunodeficient mice with hindlimb ischemia.^290^ Currently, three primary approaches are employed for utilizing EPCs in treating endothelial injury: (1) Transplanting EPCs to the site of endothelial injury to facilitate regeneration and repair of endothelial tissues. For example, injecting EPCs into mice has markedly improved damage to sinusoidal ECs and hepatocytes, reduced the secretion of IL‐6 and TNFα, inhibited platelet activation, and enhanced liver function.^291^ (2) Introducing specific genes, such as calcitonin gene‐related peptide (CGRP), into EPCs to boost their protective effects on ECs.^292^ (3) Administering certain drugs, including low‐dose aspirin, resveratrol, rosiglitazone, pioglitazone, and evodiamine, to delay EPC senescence. For instance, the resveratrol derivative BTM‐0512 inhibits EPC senescence in diabetic rats through mechanisms involving the SIRT1–dimethylarginine dimethylaminohydrolase 2 (DDAH2)/ADMA pathway.^293^

#### 5.3.3 Nanotechnology in targeted drug delivery to the endothelium

The fields of nanomedicine and nanoscience have experienced remarkable advancements in recent years. A variety of growth factors, including VEGF, platelet‐derived growth factor (PDGF), basic fibroblast growth factor (bFGF), TGFβ, and hepatocyte growth factor, are frequently utilized to facilitate blood vessel regeneration. A VEGF nanodelivery system, comprising dextran sulfate and a polyelectrolyte complex of various polycations (such as chitosan, PEI, and poly‐l‐lysine), was characterized by particle sizes ranging from 160 to 280 nm and a zeta potential between −12.9 and 18.7 mV. All formulations exhibited markedly enhanced VEGF release over time when compared with VEGF alone (without nanoparticles).^294^ Golub et al.^295^ demonstrated increased blood vessel formation in a mouse ischemia model by employing VEGF‐loaded ∼400 nm PLGA nanoparticles, in contrast to VEGF without any carrier. Moreover, mesoporous silica nanoparticles (57 nm), with their high surface area‐to‐volume ratio and tunable pore sizes, were found to localize in the cytoplasm of HUVECs without inducing cytotoxicity, and to release bFGF over 20 days.^296^ Despite the ability of nanoscale delivery systems to enhance the sustained release of angiogenic proteins, blood vessel regeneration demands a diverse array of growth factors and enzymes. Thus, developing more sophisticated chemical and material engineering techniques is essential to ensure multiple proteins’ efficient, controlled, and timely release within the ideal timeframe.

## 6 FUTURE DIRECTIONS AND RESEARCH OPPORTUNITIES

As knowledge regarding endothelial function and its significance in CV health continues to expand, developments in nanomedicine, gene therapy, and stem cell technologies are emerging as promising strategies for improving endothelial repair and function. The early detection and monitoring of diseases have consistently been among the most sought‐after objectives for healthcare professionals. Future research is predominantly directed toward identifying novel biomarkers and creating innovative diagnostic tools. By harnessing advanced technologies, researchers may facilitate early intervention and targeted therapies, addressing prevailing vascular diseases and fostering broader CV health.

### 6.1 Identification of novel biomarkers for endothelial injury

Adhesion molecules, such as E‐selectin, ICAM‐1, and VCAM‐1, along with those involved in the coagulation cascade, notably von Willebrand factor and soluble thrombomodulin, have been extensively investigated in this regard.^297^ Severe ED is a hallmark of sepsis, with soluble fms‐like tyrosine kinase‐1 identified as one of the most promising and novel prognostic markers for this condition.^298^, ^299^ In other diseases, including diabetes mellitus,^300^ chronic kidney disease,^301^ peripheral artery disease,^297^ and heart failure,^302^ although these classic biomarkers correlate strongly with disease severity, their efficacy in predicting CV event risk remains a topic of ongoing debate.^303^ This discrepancy arises because many of these molecules are synthesized by ECs, leukocytes, and platelets, reducing their specificity. Consequently, a more targeted biomarker would be required to more precisely reflect endothelial damage.^303^, ^304^

In the past two decades, microparticles (MPs) have garnered significant attention as biomarkers for ED and as predictors of CVDs. These minute vesicles, ranging from 0.1 to 1.0 µm in diameter, are released from the plasma membranes of various cell types, including leukocytes, platelets, and ECs. MPs transport biological material from their parent cells, which is subsequently released into the bloodstream to facilitate communication with other cell types.^305^, ^306^ Studies have demonstrated that endothelial MPs (EMPs) are markedly elevated in plasma when CV risk factors, such as smoking and obesity, are present,^307^ and in numerous CV and metabolic disorders, including CADs and diabetes mellitus.^308^ EMPs, in particular, have been proposed as important tools for CV risk stratification, as well as for monitoring disease progression, severity, and the efficacy of atheroprotective therapies.^305^, ^309^, ^310^, ^311^, ^312^, ^313^

Endoglin is a homodimeric transmembrane receptor for TGFβ1 and TGFβ3, predominantly located in the vessel wall, particularly in proliferating ECs.^313^, ^314^ Blann et al.^315^ were the first to demonstrate that soluble endoglin (sEng) levels were elevated in patients with atherosclerosis, especially in those with peripheral artery disease, and positively correlated with total cholesterol levels. However, it is crucial to note that the findings were inconsistent across various studies, which may undermine the reproducibility and applicability of sEng as a biomarker for ED. Further research is required to better elucidate the role of sEng in CVDs. Additionally, endocan, a soluble dermatan sulfate proteoglycan, is primarily secreted by vascular ECs.^316^ Endocan expression in ECs is upregulated in response to inflammatory stimuli,^317^ leading to elevated serum endocan levels in autoimmune and systemic inflammatory diseases. This has been confirmed in studies on psoriasis,^318^ systemic sclerosis,^319^ systemic lupus erythematosus,^320^ sepsis,^321^ and acute respiratory distress syndrome.^322^ While most studies linking endocan to vascular disease are cross‐sectional, they consistently suggest that endocan could serve as a robust and reliable marker of ED.

### 6.2 Development of diagnostic tools

Evaluating vascular endothelial function is pivotal in the early detection, diagnosis, risk stratification, treatment, therapeutic monitoring, and long‐term prognosis of CVDs. Such assessments are instrumental in enhancing both quality of life and living standards across populations. Over recent years, there has been a significant shift in the methods used to evaluate vascular endothelial function, evolving from invasive techniques to noninvasive approaches.

The assessment of endothelial function entails evaluating the responsiveness of ECs to stimuli that induce vasodilation or vasoconstriction. As our understanding of ED deepens, this evaluation is increasingly recognized as an essential tool for monitoring disease progression and as a potential clinical marker for CV complications, even in individuals without overt symptoms. Invasive techniques allow for the direct measurement of vasodilation in response to both endothelium‐dependent and endothelium‐independent stimuli. For example, coronary angiography with acetylcholine is regarded as the gold standard for assessing coronary endothelial function. Research has established that the intracoronary administration of acetylcholine in patients with coronary atherosclerotic heart disease (commonly termed coronary artery disease, CAD) serves as an independent predictor of CAD progression and long‐term CV events. This approach enhances the diagnostic accuracy of CAD and assists in evaluating the long‐term prognosis of patients. Additionally, the coronary microvascular resistance index is considered a reliable indicator for clinically assessing coronary microvascular endothelial function. However, both techniques remain invasive, associated with high detection thresholds, time‐consuming procedures, and considerable costs.^323^, ^324^, ^325^

Noninvasive techniques for evaluating endothelial function have seen broader clinical adoption owing to their noninvasive nature and simplicity. The most commonly utilized methods include FMD and reactive hyperemia‐peripheral arterial tonometry (RH‐PAT). FMD primarily focuses on assessing endothelial function in larger vessels, such as peripheral and coronary arteries, while RH‐PAT is mainly employed for evaluating endothelial function in smaller vessels (diameter ≤ 200 µm).^326^, ^327^, ^328^, ^329^ Other less frequently used methods, such as photoplethysmography and digital thermal monitoring, have been reported. Elevated FMD values are indicative of improved endothelial function and reduced arterial stiffness. The FMD procedure is straightforward and rapid, requiring no separate electrocardiogram (ECG) monitoring or blood pressure measurements. It allows for the real‐time tracking of the maximum vessel diameter following dilation, thereby markedly reducing the measurement time.^326^, ^327^ Research has demonstrated that the RH‐PAT method strongly correlates with the “gold standard” approach. Using a reference reactive hyperemia index (RHI) threshold of ≥1.67, a higher RHI indicates superior endothelial function, whereas an RHI below 1.67 suggests the presence of ED.^329^ The RH‐PAT method addresses some of the limitations of FMD, such as operator dependency, the absence of standardized measurement protocols, and inconsistent criteria. It provides advantages like noninvasiveness, safety, reproducibility, and fully automated analysis. However, several studies have produced conflicting results. Matsuzawa et al.^330^ reported a significant correlation between flow‐dependent vasodilation, as measured by FMD and RH‐PAT, while Lee et al.^331^ argued that no such correlation exists and raised concerns regarding the validity of PAT.

## 7 CONCLUSION

This review comprehensively analyzes the causes, mechanisms, and therapeutic approaches related to EC injury. It specifically examines the molecular pathways underlying endothelial damage, including OS, mitochondrial dysfunction, inflammation, immune responses, and endothelial permeability and barrier function alterations. Additionally, the review emphasizes the critical involvement of endothelial injury in the pathogenesis and progression of diseases such as cardiac I/R injury, sepsis, and vascular complications associated with diabetes.

EC injury arises due to various factors, which activate OS, mitochondrial signaling pathways, and inflammatory responses, leading to changes in the molecular structure of EC surfaces, thereby contributing to ED and apoptosis. Under pathological conditions, disruptions in mitochondrial dynamics, including imbalances between fission and fusion, as well as impairments in mitophagy and biogenesis, result in augmented ROS production and ATP depletion. ROS, functioning as second messengers, convey physiological signals downstream, initiating OS, inflammation, and cellular component damage. Inflammatory responses are orchestrated through multiple molecular pathways, with NF‐κB, HMGB1, and inflammasomes serving as pivotal regulators. Moreover, inflammatory mechanisms are modulated by specific miRNAs, while EC senescence further intensifies the inflammatory response. External stimuli induce the shedding of the endothelial glycocalyx, which exposes leukocyte receptors, thereby facilitating leukocyte adhesion. Furthermore, glycocalyx components themselves possess proinflammatory properties. These stimuli also trigger the reorganization of EC junctions, which increases permeability and causes vascular leakage.

The current therapeutic approaches to EC injury primarily aim to restore endothelial function by applying antioxidants, anti‐inflammatory agents, and medications that stimulate NO production. Additionally, novel strategies such as stem cell therapy, transplantation of EPCs, and nanotechnology for targeted drug delivery are being explored to facilitate the repair of damaged endothelium. Lifestyle modifications, including dietary adjustments and physical activity, are also integral to supporting endothelial health. Concurrently, with the ongoing advancement of technology and methodologies, the capacity for the early detection and diagnosis of EC injury is progressively improving. Several advanced imaging techniques and biomarker technologies have been described, which enhance real‐time insight into endothelial dynamics, thus promoting early diagnosis and the development of personalized treatment regimens. Treatments targeting EC injury are continually evolving, with ongoing refinements and testing. Although challenges and controversies persist, promising results have already been observed.

In conclusion, EC injury is essential in the pathogenesis of numerous CV and cerebrovascular diseases. Early detection of EC injury and timely intervention are paramount for addressing clinical challenges; however, existing detection and treatment strategies remain limited. Furthermore, the advancement of detection and therapeutic approaches targeting EC injury holds great promise for improving patient outcomes and represents a significant step forward in medical research.

## AUTHOR CONTRIBUTIONS

Tian Xia contributed to the data curation, investigation, and writing original draft. Jiachi Yu contributed to the data curation, investigation, and validation. Meng Du contributed to the validation and editing. Ximeng Chen contributed to the proofreading and editing. Chengbin Wang contributed to the conceptualization and project administration. Ruibing Li contributed to the conceptualization, project administration, resources, and supervision. All authors contributed to reviewing and editing the manuscript. All authors have read and approved the final manuscript.

## CONFLICT OF INTEREST STATEMENT

The authors declare no conflicts of interest.

## ETHICS STATEMENT

Not applicable.

## Data Availability

Not applicable.
